# Artificial Intelligence in Sub-Elite Youth Football Players: Predicting Recovery Through Machine Learning Integration of Physical, Technical, Tactical and Maturational Data

**DOI:** 10.3390/healthcare13243301

**Published:** 2025-12-16

**Authors:** Pedro Afonso, Pedro Forte, Luís Branquinho, Ricardo Ferraz, Nuno Domingues Garrido, José Eduardo Teixeira

**Affiliations:** 1 Biosciences Higher School of Elvas, Polytechnic Institute of Portalegre, 7300-110 Portalegre, Portugal; luisbranquinho@ipportalegre.pt; 2 Department of Sport, Exercise and Health Sciences, University of Trás-os-Montes e Alto Douro, 5000-801 Vila Real, Portugal; 3 Research Centre for Active Living and Wellbeing (Livewell), Instituto Politécnico de Bragança, 5300-253 Bragança, Portugal; pedromiguelforte@gmail.com; 4 Department of Sports, Higher Institute of Educational Sciences of the Douro, 4560-708 Penafiel, Portugal; 5 CI-ISCE, ISCE Douro, 4560-547 Penafiel, Portugal; 6 Department of Sports Sciences, Instituto Politécnico de Bragança, 5300-252 Bragança, Portugal; 7 Life Quality Research Center (LQRC-CIEQV), 2001-964 Santarém, Portugal; 8 Department of Sport Sciences, University of Beira Interior, 6201-001 Covilhã, Portugal; 9 Research Center in Sports Sciences, Health Sciences and Human Development (CIDESD), 5000-801 Vila Real, Portugal; 10 Department of Sports Sciences, Polytechnic Institute of Guarda, 6300-559 Guarda, Portugal; jose.eduardo@ipg.pt; 11 Department of Sports Sciences, Polytechnic of Cávado and Ave, 4750-810 Guimarães, Portugal; 12 SPRINT—Sport Physical Activity and Health Research & Innovation Center, 6300-559 Guarda, Portugal

**Keywords:** machine learning, youth soccer, recovery, maturation, performance

## Abstract

**Background:** Monitoring training load and recovery is essential for performance optimization and injury prevention in youth football. However, predicting subjective recovery in preadolescent athletes remains challenging due to biological variability and the multidimensional nature of training responses. This exploratory study examined whether supervised machine learning (ML) models could predict Total Quality of Recovery (TQR) using integrated external load, internal load, anthropometric and maturational variables collected over one competitive microcycle. **Methods:** Forty male sub-elite U11 and U13 football players (age 10.3 ± 0.7 years; height 1.43 ± 0.08 m; body mass 38.6 ± 6.2 kg; BMI 18.7 ± 2.1 kg/m^2^) completed a microcycle comprising four training sessions (MD-4 to MD-1) and one official match (MD). A total of 158 performance-related variables were extracted, including external load (GPS-derived metrics), internal load (RPE and sRPE), heart rate indicators (U13 only), anthropometric and maturational measures, and tactical–cognitive indices (FUT-SAT). After preprocessing and aggregation at the player level, five supervised ML algorithms—K-Nearest Neighbors (KNN), Support Vector Machine (SVM), Decision Tree (DT), Random Forest (RF), and Gradient Boosting (GB)—were trained using a 70/30 train–test split and 5-fold cross-validation to classify TQR into Low, Moderate, and High categories. **Results:** Tree-based models (DT, GB) demonstrated the highest predictive performance, whereas linear and distance-based approaches (SVM, KNN) showed lower discriminative ability. Anthropometric and maturational factors emerged as the most influential predictors of TQR, with external and internal load contributing modestly. Predictive accuracy was moderate, reflecting the developmental variability characteristics of this age group. **Conclusions:** Using combined physiological, mechanical, and maturational data, these ML-based monitoring systems can simulate subjective recovery in young football players, offering potential as decision-support tools in youth sub-elite football and encouraging a more holistic and individualized approach to training and recovery management.

## 1. Introduction

The continuous monitoring of training load and recovery is fundamental for optimizing performance and preventing injuries in youth football athletes, who exhibit high variability in physiological, mechanical, and cognitive responses due to differences in maturation, anthropometry, and technical-tactical understanding [1,2]. Individualized monitoring strategies that account for these developmental differences are essential, incorporating tools to assess both physiological and psychological parameters [1] and implementing age-appropriate, engaging injury prevention protocols [3,4]. Effective programs should integrate coach education and athlete feedback to support physical and emotional well-being [5]. Comprehensive monitoring must include external loads, perceived exertion, and recovery data to provide a holistic understanding of athlete readiness and injury risk, enabling evidence-based, adaptive training interventions [6,7,8]. Moreover, contextual and interpersonal factors such as coaching style and social support play critical roles in athlete safety and development [9]. Ultimately, the interplay between individualized load monitoring, age-related physiological responses, and systematic injury prevention highlights the complexity of managing youth football athletes, underscoring the need for continued research addressing maturation-related differences to sustain performance optimization and injury reduction [7,10].

Integrated load monitoring frameworks have recently emerged to unify diverse data types, combining external loads (e.g., GPS-derived mechanical metrics) with internal loads (e.g., heart rate and rating of perceived exertion, RPE) and tactical-cognitive performance indicators. This allows the generation of comprehensive datasets supporting athlete development [11,12]. However, interpreting such multidimensional information in preadolescent athletes is challenging due to non-linear interactions between growth, training workload, and recovery [13,14]. Accurate interpretation is essential, as youth maturation variability can obscure load–response relationships, and excessive workloads during growth spurts may heighten injury risk [12,14,15]. Therefore, tailored and individualized monitoring strategies are required to adapt training regimens, reducing overload and accounting for athletes’ unique physiological and recovery responses [16,17,18,19]. Future research should explore the complex interplay among load components, physiological responses, and psychosocial factors, potentially leveraging machine learning to predict performance and injury outcomes [20,21,22]. Such advancements would enable more effective and individualized coaching strategies, optimizing athlete welfare and long-term development in demanding youth sports contexts like football [23,24,25,26].

The Total Quality of Recovery (TQR) scale has emerged as a reliable subjective indicator of an athlete’s psychophysiological readiness to perform, showing correlations with training load measures such as session-RPE (sRPE) and heart rate variability (HRV) in both adolescent and adult athletes [8,27]. By integrating psychological and physiological markers, TQR offers a multidimensional perspective on recovery, encompassing factors such as nutrition, hydration, sleep, rest, and physical activity [28]. However, in younger athletes, these relationships appear less consistent due to developmental and perceptual differences that influence fatigue perception and recovery appraisal [29,30]. Traditional physiological metrics may not fully capture the complex interplay of growth, maturation, and psychological stress in youth athletes, which can lead to incomplete recovery and increased injury risk under high training loads [31,32]. Therefore, a deeper understanding of TQR’s applicability to younger populations is necessary to design age-appropriate recovery and training protocols that support safe and sustainable athletic development [33].

Recent advances in machine learning (ML) have introduced powerful tools for analyzing complex, non-linear relationships in sports performance data, enabling the identification of hidden patterns across physiological and behavioral variables to predict readiness, recovery, and performance outcomes [34]. These techniques have proven effective in forecasting match outcomes, injury risk, and workload responses in senior athletes [35,36], yet their application in youth football, particularly regarding subjective recovery measures, remains limited. Challenges arise from developmental differences that affect how young athletes perceive and report recovery, as well as from the psychological and physiological variability inherent to this group [37,38,39]. Youth athletes’ lower developmental maturity may further hinder accurate self-assessment, complicating ML models built primarily on adult data [40,41]. Given the non-linear responses of young athletes to training loads, ML systems must be specifically adapted to account for growth-related variability and perceptual differences [35,39,42]. Overall, while ML holds great potential for improving recovery and performance monitoring, its effective implementation in youth sports demands tailored, developmentally sensitive approaches supported by continued research [43]. These developmental and perceptual instabilities represent a fundamental limitation for ML applications in youth sports, as they reduce signal consistency, increase inter-individual noise, and challenge the reliability of predictive models built on small or heterogeneous samples [44]. As a result, ML approaches validated in adult or elite populations cannot be directly transferred to pre-adolescents without substantial adaptation. This limitation further reinforces the need for age-specific modeling frameworks and highlights the scientific relevance of investigating ML-based recovery prediction in U11 and U13 players.

In parallel with these challenges, the recent literature on load monitoring for young athletes highlights the need for methodologies capable of integrating physical, technical, tactical, and maturational variables to predict subjective recovery states in pre-adolescent players. However, a clear literature gap remains for younger competitive categories, particularly U11 and U13, where developmental heterogeneity is greatest. These ages encompass the initial phases of rapid biological and cognitive maturation, during which perceptual abilities, coordination, and tactical understanding are still consolidating [45,46,47]. While most existing studies focus on adolescent groups (≥U14), early developmental categories remain understudied despite presenting unique challenges: highly variable maturation rates, greater sensitivity to training stress, and less stable subjective perception of recovery [46,48,49]. Investigating U11 and U13 athletes is thus crucial to understanding how growth-related changes influence training tolerance and recovery dynamics. These age groups also represent a stage in which technical and tactical skills are in accelerated development, potentially altering the interaction between load, decision-making, and fatigue [50,51]. Gaining insight into these processes can improve age-appropriate training strategies, support injury reduction, and optimize developmental pathways during the formative years of football participation [52,53]. The need to explore these dynamics reinforces the relevance of targeted research involving U11 and U13 athletes, particularly using integrated and data-driven approaches.

This study aimed to apply ML models to predict TQR based on physiological, mechanical, and maturational variables in sub-elite U11 football players. The specific objectives were to examine correlations between recovery and selected internal, external, and tactical performance metrics, evaluate the predictive accuracy of five supervised ML algorithms, and identify the most influential predictors of perceived recovery. By integrating anthropometric, training load, and cognitive–tactical dimensions, this exploratory analysis sought to enhance the understanding of recovery mechanisms in developing athletes and assess the feasibility of data-driven modeling for monitoring recovery in youth football. This study hypothesized that supervised machine learning models could predict the TQR in sub-elite under-11 football players using integrated physiological, mechanical, and maturational variables. It was further expected that non-linear, tree-based algorithms would achieve higher predictive accuracy than linear models and that maturational and anthropometric factors would emerge as the most influential predictors of perceived recovery.

## 2. Materials and Methods

### 2.1. Study Design

This observational and longitudinal study was carried out over the 2024/2025 season and focused on monitoring training and match performance among under-11 (U11) and under-13 (U13) sub-elite football players. The primary aim was to quantify, integrate, and model physiological, mechanical, and tactical variables gathered during training sessions and official matches. These variables provided a multidimensional framework that combined external load (GPS-derived metrics), internal load (heart rate and perceived exertion), and tactical–cognitive indicators (decision-making and positioning indices).

The monitored microcycle comprised four consecutive training sessions (MD-4, MD-3, MD-2, MD-1) and one official match (MD). Each 90 min session was conducted on outdoor synthetic turf pitches compliant with FIFA standards (100 × 70 m). Training loads were individualized according to players’ developmental stages to ensure age-appropriate progression and recovery. Environmental conditions were stable throughout the study, with temperatures ranging from 14 to 20 °C and relative humidity between 52 and 66%. All sessions were held between 4:00 p.m. and 8:00 p.m., minimizing heat stress and maintaining consistent conditions.

Training microcycles were collaboratively designed with the coaching and performance staff to align with the team’s developmental objectives. Each session began with a standardized warm-up protocol comprising (1) 5–7 min of low-intensity running, (2) dynamic stretching for the lower limbs, (3) technical passing and ball control drills, and (4) small-sided games and possession games. This consistent structure ensured comparability across microcycles and accurately reflected the athletes’ habitual training demands within a realistic competitive environment.

**Figure 1:**
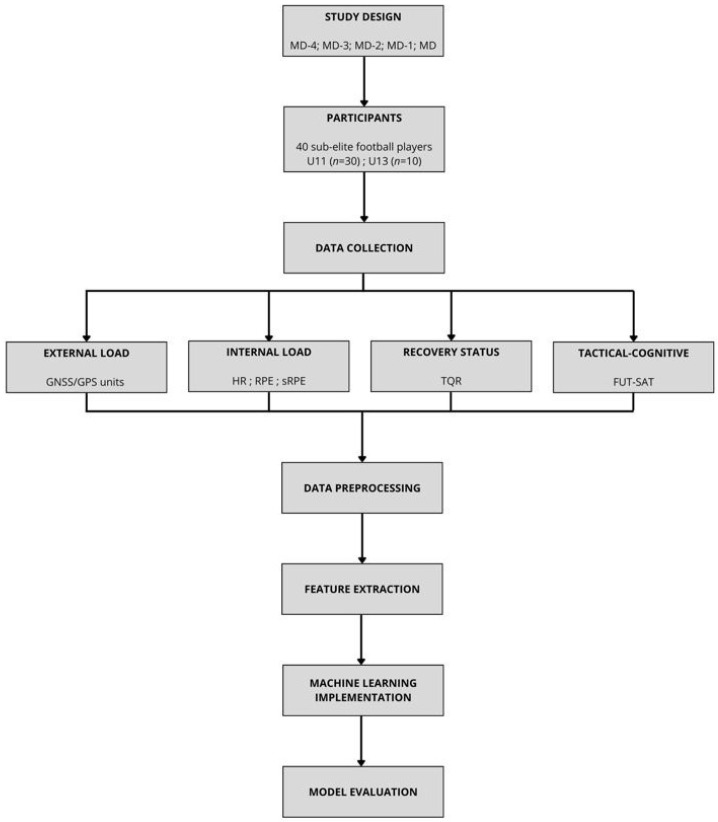
Flowchart about participant recruitment, monitoring procedures, data preprocessing, feature extraction, and machine learning implementation used to predict TQR model evaluation. Note: Global Navigation Satellite System (GNSS) and Global Positioning System (GPS); Heart Rate (HR); Rating Perception Effort (RPE); Session Rating Perception Effort (sRPE). Bold terms are the evaluated dimensions.

An overview of the study design, data collection procedures, preprocessing steps, and machine learning workflow is presented in Figure 1.

### 2.2. Participants

The sample consisted of 40 male sub-elite youth football players competing in the U11 and U13 category of a Portuguese regional football club. The players’ anthropometric characteristics were as follows (mean ± SD): age 10.3 ± 0.7 years, height 1.43 ± 0.08 m, body mass 38.6 ± 6.2 kg, and body mass index (BMI) 18.7 ± 2.1 kg/m^2^. All participants had a minimum of four years of structured football experience and trained regularly four times per week, in addition to participating in one official match per week. Eligibility criteria included: (i) participation in a competitive week including one official match; (ii) completion of all four training sessions and the match; and (iii) absence of medical conditions or injuries that could interfere with data collection. Exclusion criteria comprised: (i) missing any training session; (ii) unavailability for the match; (iii) incomplete GPS, heart rate (HR), RPE, or TQR data; and (iv) injury during the observation period.

All procedures complied with the Declaration of Helsinki (2013 revision). Prior to participation, players and their legal guardians received detailed information about the study’s aims, procedures, and potential risks. Participation was voluntary, with written informed consent obtained from parents or legal guardians and verbal assent from the players. Ethical approval was granted by the Ethics Committee of the University of Trás-os-Montes e Alto Douro (Doc104-CE-UTAD-2025). Data confidentiality was ensured through anonymization using player identification codes, and only aggregated results were reported to protect individual privacy.

### 2.3. Data Collection

The study combined technological monitoring systems with subjective self-report instruments to capture a comprehensive view of each player’s physical and cognitive performance.

#### 2.3.1. External Load Monitoring

The quantification of external load was conducted using STATSports Apex^®^ GNSS/GPS units (v. 2.0, Newry, Northern Ireland, UK), which tracked players’ positional and kinematic data throughout all training sessions and matches. Each device collected positional information at a sampling rate of 18 Hz, complemented by accelerometer (100 Hz), magnetometer (10 Hz), and gyroscope (100 Hz) sensors to capture multidimensional motion data. Players wore the sensors in a customized vest that positioned the device between the scapulae to ensure stable signal reception. Before each session, devices were activated at least 30 min in advance to guarantee accurate satellite synchronization, requiring a minimum of eight satellite connections for optimal tracking reliability [54].

The following external load variables were analyzed: total distance covered (TD, m), high-speed running distance (HSR, >18 km·h^−1^), high-intensity distance (HID, >21 km·h^−1^), relative distance (m·min^−1^), number of sprints (NSPR), maximum running speed (MRS, m·s^−1^), accelerations (ACC, >2 m·s^−2^), and decelerations (DEC, <−2 m·s^−2^). These metrics provide a comprehensive overview of both the volume and intensity components of physical performance during football-specific activities. The STATSports Apex^®^ technology used in this study has previously been validated for team sports, demonstrating excellent accuracy and reliability, with measurement errors typically below 2% for 10–18 Hz models [55,56,57]. Accordingly, these devices are considered an industry standard for capturing external load indicators in youth and professional football settings.

#### 2.3.2. Internal Load Monitoring

The internal load was assessed using both objective physiological and subjective perceptual indicators, allowing for a comprehensive evaluation of the players’ internal responses to training and match demands. For the objective component, HR data were collected exclusively from the U13 cohort using the Polar Team Pro^®^ system (Polar Electro, Kempele, Finland). This system continuously recorded HR throughout all sessions and official matches, enabling the analysis of the average heart rate, peak heart rate, and the distribution of time spent in five individualized intensity zones (Z1–Z5), expressed as a percentage of each player’s maximum heart rate (%HRmax). The %HRmax reference was determined from the highest HR value observed during the monitoring period for each athlete. These metrics were subsequently processed for each session and summarized across the microcycle to depict intensity fluctuations throughout the week. The U11 group was excluded from HR monitoring due to methodological limitations and comfort considerations related to chest strap fitting and data consistency in younger players.

The subjective internal load was determined through the Borg CR10 scale (0–10 points) of RPE [58]. Approximately 30 min after every training session and match, players were interviewed individually by the investigator using a digital form administered on a tablet (Google Forms^®^ interface, Google, Mountain View, CA, USA). The scale featured both numerical anchors and expressive emojis to facilitate understanding among younger athletes. Participants verbally communicated their perceived effort, which was immediately logged into the form. All players had previously undergone familiarization sessions with the RPE protocol to ensure comprehension and reliability.

Sessional training load (sRPE) was calculated using the formula sRPE = RPE × session duration (min), and the resulting values were expressed in arbitrary units (AU) [59]. The compiled data were exported to Microsoft Excel^®^ (Version 2510, Microssoft, Redmond, WA, USA) for storage, organization, and subsequent statistical analysis. The RPE and sRPE methods have been validated in youth and elite football populations as effective, non-invasive tools to monitor psychophysiological load [60,61].

#### 2.3.3. Recovery Status

Recovery status was assessed with the TQR scale (1–10), an instrument validated for youth football populations [8]. TQR was assessed 30 min before sessions to gauge readiness and recovery, allowing players to consider their physical and psychological states comprehensively [62]. This combined use of RPE (as internal load monitoring) and TQR provides an effective, low-cost framework for monitoring the interaction between training load and recovery, aligning with evidence that supports the inclusion of subjective metrics to complement objective monitoring in youth sports [63,64]. By systematically applying these psychophysiological tools, coaches can tailor training stimuli to the individual recovery profiles of young athletes, fostering improved performance, reduced injury risk, and enhanced overall well-being [65].

#### 2.3.4. Tactical-Cognitive Performance

The assessment of technical–tactical performance was carried out using the Football Tactical Assessment System (FUT-SAT), a validated and widely used instrument designed to evaluate players’ understanding and execution of tactical principles in football [66,67]. This protocol enables systematic observation and quantification of both offensive and defensive game principles in representative playing contexts, providing a holistic understanding of the players’ decision-making and motor execution capacities. The inclusion of FUT-SAT in this study provides a robust framework for linking technical–tactical behavior with training load and recovery dynamics. By analyzing decision quality (DMI) and execution efficiency (MEI), the study captures how players’ cognitive and motor processes interact under varying physiological demands. This multidimensional approach aligns with contemporary research emphasizing the integration of physical, psychological, and tactical domains in football performance analysis [68,69]. 

The FUT-SAT protocol was implemented through small-sided games (SSGs) with the format GK + 3 vs. 3 + GK, which provides an ecologically valid and developmentally appropriate environment for tactical assessment in youth football [70]. Each SSG was played on a 36 × 27 m pitch, maintaining proportional field dimensions that ensure representativeness of official play conditions for young athletes [71]. Every game lasted 4 min, during which players were encouraged to maintain competitive intensity and apply game principles naturally.

The SSGs were video recorded using a GoPro HERO Action Camera (4K, 12 MP, Wi-Fi, Bluetooth) positioned at an elevated angle to capture all playing actions. Recorded footage was subsequently analyzed using LongoMatch^®^ software (LongoMatch^®^ version 1.5.9, Barcelona, Spain), which allows frame-by-frame annotation of game events and precise tagging of tactical actions for subsequent coding and quantification [72].

Player actions were analyzed and categorized based on the core tactical principles defined in the FUT-SAT model, which encompasses both offensive and defensive dimensions of play. The offensive principles included penetration, offensive coverage, mobility, and the effective use of space, while the defensive principles comprised delay, defensive coverage, balance, and concentration. Each action performed by a player was independently evaluated according to two analytical dimensions: decision-making, which assessed whether the player’s tactical decision was appropriate or inappropriate within the specific game context, and motor execution, which determined whether the player’s technical action was effective or ineffective in carrying out the intended decision. From these assessments, two main performance indicators were derived. The DMI represented the proportion of correct tactical decisions relative to the total number of decisions made, and the MEI quantified the proportion of technically successful executions relative to all attempted actions. Both indexes ranged from 0 to 1, with higher values reflecting greater tactical intelligence and execution quality. Additionally, an Overall Performance Indicator (OPI) was computed to integrate these dimensions, providing a comprehensive representation of each player’s tactical behavior by combining their decision-making and execution efficiency across all offensive and defensive principles [67], as presented in equation

(1) $$OPI=\frac{\sum (DMI+MEI)}{n}$$

where *n* represents the number of analyzed tactical principles. This composite indicator offers a global assessment of tactical efficiency during game performance, facilitating comparisons between individuals or groups (U11 vs. U13).

To ensure objectivity and consistency, all video analyses were performed by two independent researchers previously trained in the FUT-SAT coding system. A familiarization phase was conducted using pilot videos not included in the study sample. Inter- and intra-observer reliability were assessed through Cohen’s kappa (κ) coefficients, yielding values above 0.85 for both decision-making and motor execution variables, which is considered excellent agreement [73].

### 2.4. Data Preprocessing and Normalization

Data processing and analysis were conducted using Python™ (version 3.10.4, Centrum Wiskunde & Informatica (CWI) in Amsterdam, The Netherlands), employing widely recognized libraries such as pandas, numpy, matplotlib, and scikit-learn for efficient data manipulation, visualization, and model preparation. Prior to analysis, data quality was ensured through several preprocessing steps. Missing values, which represented less than 10% of the dataset, were imputed using the mean of each respective variable to preserve data integrity while minimizing bias. Outliers exceeding ±3 standard deviations from the mean were identified and removed to prevent distortion of model performance. Categorical variables were transformed into numerical representations using one-hot encoding, allowing for their inclusion in machine learning algorithms that require numerical inputs. Continuous variables were then standardized using the StandardScaler function from sklearn, preprocessing, normalizing each feature to have a mean of zero and a standard deviation of one. This step was crucial for ensuring feature comparability and preventing scale-related bias, thereby improving the stability and interpretability of the subsequent machine learning models.

A total of 158 performance-related variables were computed across all monitoring domains (Table 1). These included: (i) external load metrics derived from GPS data (e.g., total distance, high-speed running, accelerations, decelerations, and peak speed); (ii) internal load indicators obtained from RPE, sRPE and HR-derived metrics; (iii) anthropometric and maturational variables (e.g., height, leg length, body mass, maturity offset); and (iv) tactical–cognitive indices derived from FUT-SAT observations. The aim of generating such a comprehensive dataset was to capture the full multidimensional profile of each player, allowing machine learning algorithms to explore non-linear interactions among physical, physiological, perceptual, and tactical factors. For the purposes of machine learning modeling, data from one competitive microcycle (four training sessions: MD-4, MD-3, MD-2, MD-1, and one official match: MD) were aggregated at the player level. Thus, the final dataset consisted of 40 complete observations (one per player), each observation including 158 performance-related variables describing that player’s external load, internal load, anthropometric/maturational profile, and tactical–cognitive performance across the microcycle.

### 2.5. Machine Learning Implementation

A supervised machine learning analysis was conducted to examine relationships between physical, physiological, and cognitive variables. The dataset was split into training (70%) and testing (30%) subsets using a random seed of 42 for reproducibility. Five classification algorithms were applied: K-Nearest Neighbors (KNN), based on distance between data points; Support Vector Machine (SVM), which separates classes using hyperplanes; Random Forest (RF), an ensemble of decision trees designed to reduce overfitting; Decision Tree (DT), a rule-based hierarchical classifier; and Gradient Boosting (GB), an ensemble boosting method that builds additive decision-tree models to improve predictive accuracy by sequentially reducing residual errors. Model training was performed using scikit-learn v1.3 (Python™, version 3.10.4, Centrum Wiskunde & Informatica (CWI) in Amsterdam, The Netherlands), with a 5-fold cross-validation procedure applied to obtain stable performance estimates and minimize variance. All algorithms were trained using their standard default hyperparameter configurations, following established guidelines for exploratory modeling in small datasets. This approach ensured methodological consistency across models while avoiding overfitting that may arise from excessive tuning in limited samples.

Model performance was assessed using accuracy, precision, recall, and F1-score metrics computed with the classification_report function. This exploratory analysis aimed to identify potential predictors of internal load and tactical indices rather than to establish definitive predictive systems.

### 2.6. Statistical Analysis

Descriptive statistics (mean ± SD) were computed for all quantitative variables. Normality was assessed with the Shapiro–Wilk test, and homogeneity of variances was tested using Levene’s test. Differences between training days (MD-4, MD-3, MD-2, MD-1) and match day (MD) were analyzed using one-way repeated-measures ANOVA, with Bonferroni post hoc correction when necessary. For non-normal data, the Friedman test was applied. Effect sizes were calculated using Cohen’s d and interpreted as small (0.2), moderate (0.5), or large (0.8). Correlations between internal and external load indicators, as well as between physical and tactical indices, were evaluated using Pearson’s or Spearman’s correlation coefficients depending on data distribution. All statistical analyses were performed using Python 3.10.4 and IBM SPSS Statistics v.29 (Armonk, NY, USA), with the level of significance set at *p* < 0.05.

## 3. Results

### 3.1. Variable Selection for the ML Algorithm

To ensure that the model effectively predicts TQR, a variable selection procedure was performed combining domain knowledge, correlation analysis, and statistical significance testing. Although 158 variables were initially extracted, only 17 were retained for modeling. The reduction was performed through a multi-step procedure combining: (i) theoretical relevance to recovery monitoring; (ii) correlation analysis to remove redundant highly collinear features; (iii) data completeness across all sessions; and (iv) preliminary feature screening using tree-based importance metrics. This process ensured that only the most informative and non-redundant predictors were entered in the final ML models. The selected variables were grouped into four main categories:

1. Technical and tactical performance—*MEI_Total_Index*, *DMI_Total_Index*, and *Performance_MEI_DMI* (sum of MEI and DMI);
2. Internal training load—*Mean HR%, Max HR%, % Time Zone 1–5, sRPE_1–4* and *sRPE_MD*;
3. External training load—*ACC*, *DEC*, *HSR*, *Distance*, and *Number of Sprints*;
4. Anthropometric and maturational variables—*Height*, *Weight*, *Leg Length*, and *Mirwald Maturity Offset*.

No statistically significant correlations were identified (*p* > 0.05) with TQR. However, all features were retained in the models, given their theoretical and physiological relevance to recovery and fatigue monitoring. This approach allowed the algorithms to capture potential non-linear and multivariate dependencies that are not detectable through linear correlation analysis.

### 3.2. Algorithm Performance, Feature Importance, and Predictive Accuracy

Five supervised learning algorithms were implemented to classify TQR into three recovery categories (*Low*, *Medium*, and *High*): (1) KNN, (2) SVM, (3) DT Classifier, (4) RF Classifier, and (5) GB Classifier.

These models were selected because they represent a diverse range of linear vs. non-linear, parametric vs. non-parametric, and ensemble vs. single-tree approaches. Tree-based models (RF and GB) were included for their robustness in capturing hierarchical and interaction effects between multiple training and anthropometric variables.

All models were trained using 70% of the dataset and evaluated on the remaining 30%, with 5-fold stratified cross-validation applied to the training set. The metrics used for evaluation were Accuracy, Precision, Recall, F1-score, and the Average Metric (mean of the four). The results are shown in Table 2.

Among all models, the DT Classifier achieved the highest overall performance (Accuracy = 70.0%; Average Metric = 71.5%), followed closely by the GB Classifier. Tree-based models displayed superior performance compared to linear or distance-based methods, indicating that recovery perception is governed by non-linear relationships involving multiple performance and maturational factors. The feature importance extracted from the best-performing model (i.e., DT) revealed that anthropometric and maturational features contributed most to model accuracy, followed by internal training load and locomotor variables. The ten most influential predictors are presented in Table 3.

These variables collectively explain approximately 40% of the model’s variance in predicting recovery status. Thus, the DT model relies primarily on maturational and anthropometric indicators, complemented by session-based internal and external load metrics, to estimate recovery perception.

### 3.3. Model ROC/AUC

The discriminative performance of each model was assessed using Receiver Operating Characteristic (ROC) analysis (Table 4). The Decision Tree Classifier demonstrated the best discriminative ability with an *Area Under the Curve (AUC)* of 0.40, followed by the SVM (0.38) and GB (0.35). The RF and KNN models achieved lower AUC values (0.32 and 0.30, respectively).

**Figure 2:**
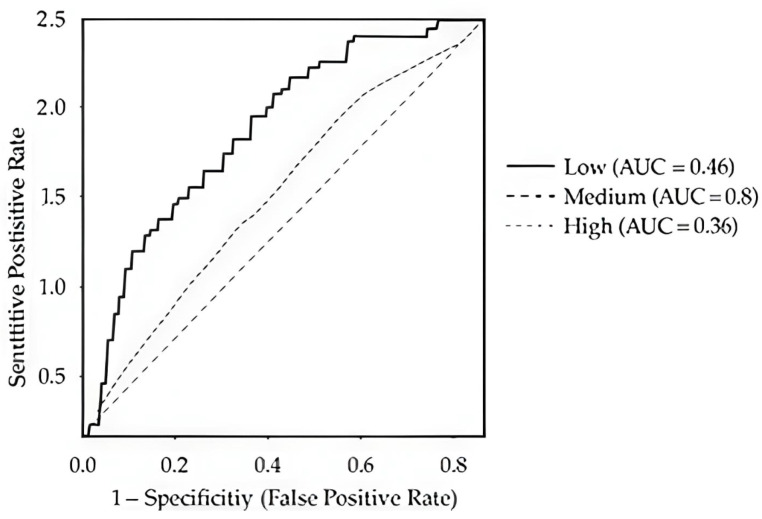
ROC curves for the DT Classifier.

**Figure 3:**
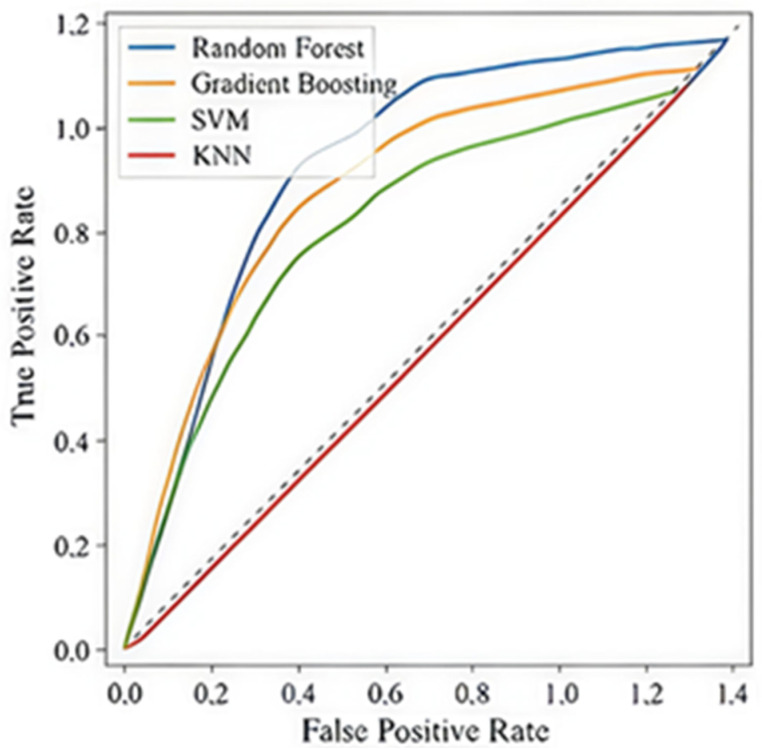
ROC curves across all algorithms.

Figure 2 presents the ROC curves for the Decision Tree Classifier, showing limited discrimination among recovery classes (*Low*, *Medium*, *High*) with both macro- and micro-average AUC = 0.40. Figure 3 compares the ROC curves across all algorithms, confirming the Decision Tree’s relative advantage.

Although all models showed modest AUC values (<0.5), their consistent ranking supports the robustness of the findings. The results indicate that recovery perception (TQR) in this sample is only partially predictable from biomechanical and physiological features, likely due to the small sample size (*n* = 40) and the subjective nature of perceived recovery. Nevertheless, the non-linear tree-based methods were better able to extract underlying patterns than linear approaches.

## 4. Discussion

The aim of this study was to apply supervised ML techniques to predict the TQR in sub-elite U11 and U13 football players based on physiological, mechanical, and maturational variables. It was hypothesized that ML models would be able to predict TQR with moderate accuracy, that non-linear, tree-based algorithms (such as DT and GB) would outperform linear approaches, and that maturational and anthropometric factors would be the most influential predictors of recovery perception. The findings partially verified this hypothesis: the DT model achieved the highest predictive accuracy (70%), confirming the superiority of non-linear algorithms, and maturational variables were the strongest contributors to model performance. However, the relatively low discriminative power (AUC ≈ 0.40) indicated that TQR was only moderately predictable, suggesting that subjective recovery in young athletes is influenced by additional physiological and psychological factors not captured in the current dataset.

The results demonstrated that the DT Classifier and GB Classifier achieved the best overall performance (Accuracy = 70%, Average Metric ≈ 71%), outperforming the SVM, RF, and KNN models. This indicates that non-linear, rule-based models are more effective in capturing the complex relationships underpinning recovery perception in young players. The ten most influential predictors identified by the Decision Tree included leg length, maturity offset, height, sRPE, and high-intensity distance, collectively explaining about 40% of model variance. These findings suggest that recovery perception is influenced not only by workload but also by biological maturation and anthropometric differences, consistent with previous research linking growth-related factors to variations in physical performance, fatigue, and recovery kinetics in preadolescent athletes. The DT and GB Classifiers achieved the highest predictive accuracy (≈70–71%), outperforming other models and highlighting the effectiveness of non-linear, rule-based algorithms in capturing complex recovery patterns in young athletes [74]. Key predictors—leg length, maturity offset, height, sRPE, and high-intensity distance—explained about 40% of model variance, showing that recovery perception depends on both training load and biological maturation [75,76,77]. These findings underscore the value of machine learning for identifying multifactorial influences on youth recovery and advocate for individualized, developmentally informed recovery strategies [78].

Interestingly, the inclusion of *tactical indices* (MEI, DMI) and *locomotor variables* (ACC, DEC, TD) contributed less to predictive accuracy than maturational features. This pattern implies that, at this developmental stage, biological readiness may play a greater role in perceived recovery than tactical or mechanical complexity. The weak linear correlations between TQR and sRPE further support this interpretation, indicating that subjective recovery may not directly scale with session intensity but rather with the player’s developmental capacity to tolerate and interpret training stress. Tactical indices such as the MEI and DMI, along with locomotor variables (ACC, DEC, TD), contributed less to recovery prediction than maturational features, suggesting that biological readiness plays a more decisive role in perceived recovery at this developmental stage [79,80]. Biological maturation factors like maturity offset and anthropometry strongly influence performance and recovery during growth phases [81,82]. The weak correlation between TQR and sRPE further indicates that subjective recovery is shaped more by developmental capacity than by training intensity [83,84,85]. Thus, incorporating biological indicators into performance assessment models can improve the understanding of youth athletes’ responses, enabling more individualized and developmentally informed recovery and coaching strategies [86].

ROC/AUC analyses showed limited discriminative ability across all models (AUC = 0.30–0.40), with the Decision Tree performing best (0.40), followed by SVM (0.38) and Gradient Boosting (0.35). Although these results indicate low classification accuracy, they are consistent with previous findings in youth sports ML research reporting similarly constrained predictive performance [87,88,89]. The results also reinforce that subjective recovery is a multifactorial construct shaped by both physiological and psychological factors [90], requiring consideration of variables such as sleep quality, nutrition, and psychological well-being [91,92]. The weak association between TQR and sRPE further suggests that subjective recovery reflects developmental capacity more than objective training intensity [93,94]. Overall, these findings highlight the need to improve predictive accuracy by integrating physiological, psychological, and contextual factors into future models of youth athlete recovery [95,96].

From a practical perspective, this study demonstrates the feasibility of ML approaches to youth football monitoring systems, highlighting their potential to support evidence-based decision-making in player management. Despite the relatively small dataset, the algorithms were capable of identifying meaningful predictors of recovery perception, suggesting that maturational factors—such as leg length, maturity offset, and height—should be monitored alongside traditional workload indicators to enhance understanding of players’ recovery dynamics and readiness to train. These findings emphasize the importance of individualized load management strategies that account for the physiological and developmental variability characteristic of preadolescent athletes. ML-driven monitoring tools could eventually aid coaches and sport scientists in the early detection of maladaptation, under-recovery, or excessive fatigue, contributing to more precise and age-appropriate training interventions. However, this potential must be interpreted within the limitations of the current study, particularly the small sample size (*n* = 40) and the subjective nature of the TQR measure, which may be affected by players’ cognitive and motivational maturity. Moreover, the absence of variables related to sleep quality, nutrition, and psychological stress may have limited the model’s predictive capacity, given the multifactorial nature of recovery. Future studies should therefore aim to collect larger, multicentric datasets encompassing a broader range of psychophysiological, contextual, and behavioral variables. Incorporating wearable-based sleep and recovery markers, mood profiling, and training context descriptors could improve the granularity and accuracy of predictive models. Additionally, the exploration of ensemble and deep learning methods, such as convolutional or recurrent neural networks, may provide superior pattern recognition capabilities, enabling the development of more robust and generalizable recovery prediction systems for youth athletes. Expanding this perspective, future research may also integrate technical–motor assessments and long-term developmental indicators, which were beyond the scope and feasibility of the present applied setting but could contribute additional predictive relevance in larger and more controlled cohorts.

## 5. Conclusions

This exploratory study provides preliminary evidence that machine learning techniques can model subjective recovery in young football players using integrated physiological, mechanical, and maturational data. Although predictive accuracy was moderate and AUC values low, the results emphasize the role of biological maturation as a key determinant of recovery perception in preadolescent athletes. The study demonstrates the potential of ML-based monitoring systems as decision-support tools in youth sport, promoting a more holistic and individualized approach to training and recovery management.

### Limitations

This study has some limitations that should be acknowledged. First, the relatively modest sample size (*n* = 40), although typical of applied research in single-club youth cohorts, limits the generalizability of the findings and may contribute to model variability. Second, data were collected over a single competitive microcycle, which limits the ability to capture week-to-week variability in training load, recovery status, and tactical–technical behavior. Third, the HR data were only available for the U13 players due to methodological and practical constraints, preventing complete physiological comparison across age groups. Fourth, while the machine learning models showed moderate predictive capacity, the absence of external validation limits the extrapolation of the results and highlights the need for replication in independent samples. Finally, natural inter-individual differences in biological maturation among preadolescent athletes may have influenced perceived recovery and contributed to model variability. Future research should incorporate larger multi-club cohorts, multi-week monitoring, full HR datasets across age groups, and external validation to strengthen model reliability and predictive robustness.

## Figures and Tables

**Table 1:** Categorisation of the 158 extracted performance-related variables across monitoring domains.

Category	Variables Included	Number of Variables
Technical and tactical performance	TD, HSR, HID, Distance per Minute, NSPR, MRS, ACC, DEC—all computed for MD-4, MD-3, MD-2, MD-1, MD.	40
Internal Training Load	HR Metrics (HR% mean, HR%max, HR Zones Z1–Z5), perceptual load (RPE, sRPE), and recovery (TQR), collected per session (MD-4 to MD).	45
External Training Load	Height, Body Mass, BMI, Sitting Height, Leg Length, CA, MO (Mirwald).	8
Anthropometric and Maturational	Offensive Principles (Penetration, Offensive Coverage, Mobility, Width/Space, Offensive Unity); Defensive Principles (Delay, Defensive Coverage, Balance, Concentration); DMI, MEI, OPI. Includes counts, correct/incorrect actions, and indices per tactical principle.	58
Total Variables Extracted	-	151 *

*Abbreviations*: ACC—Accelerations; BMI—Body Mass Index; CA—Chronological Age; DEC—Decelerations; DMI—Decision-Making Index; HID—High-Intensity Distance; HSR—High-Speed Running; HR%max—Maximum Heart Rate Percentage; HR% mean—Mean Heart Rate Percentage; HRZ1–HRZ5—Heart Rate Zones 1–5; MEI—Motor Execution Index; MD—Match Day; MO—Maturity Offset; MRS—Maximum Running Speed; NSPR—Number of Sprints; OPI—Overall Performance Indicator; RPE—Rating of Perceived Exertion; sRPE—Session Rating of Perceived Exertion; TD—Total Distance; TQR—Total Quality Recovery. * Additional composite and derived variables (e.g., aggregated weekly metrics and combined tactical indices) were computed from these raw measures and included in the feature selection pipeline, resulting in a total of 158 performance-related predictors initially considered in the modeling process.

**Table 2:** Performance of machine learning algorithms in predicting TQR categories.

Algorithm	Accuracy (%)	Precision (%)	Recall (%)	F1-Score (%)	Average Metric	Cross-Validation Accuracy (%)
DT Classifier	70.00	75.56	69.44	71.11	71.53	63.33
GB Classifier	70.00	77.78	66.67	65.56	70.00	63.33
KNN Classifier	60.00	80.95	61.11	58.89	65.24	63.33
Random Forest Classifier	60.00	70.00	58.33	57.94	61.57	73.33
Support Vector Machine Classifier	40.00	26.67	38.89	31.48	34.26	56.67

*Abbreviations:* DT—Decision Tree; GB—Gradient Boosting; KNN—K-Nearest Neighbors; RF—Random Forest; SVM—Support Vector Machine.

**Table 3:** Top 10 predictors influencing TQR classification (DT model).

Rank	Feature	Importance
1	Leg Length (*Lower Limb Length*)	0.105
2	sRPE (*sRPE_MD*)	0.084
3	Height	0.083
4	Mirwald MO	0.064
5	HID (Session 4)	0.051
6	DEC (Session 2)	0.039
7	Weight	0.031
8	DEC (Session 3)	0.025
9	TD (Session 4)	0.024
10	(Mean)	0.023

*Abbreviations:* ACC—Accelerations; DEC—Decelerations; HID—High-Intensity Distance; MO—Maturity offset; sRPE—Session Rating of Perceived Exertion; TD—Total Distance.

**Table 4:** Comparative AUC values for all ML algorithms.

Algorithm	AUC
DT Classifier	0.40
SVM	0.38
GB Classifier	0.35
RF Classifier	0.32
KNN	0.30

*Abbreviations:* AUC—Area Under the Curve; DT—Decision Tree; GB—Gradient Boosting; KNN—K-Nearest Neighbors; RF—Random Forest; SVM—Support Vector Machine.

## Data Availability

The data supporting the findings of this study are not publicly available due to privacy or ethical restrictions but can be provided by the corresponding author upon reasonable request.

## Abbreviations

The following abbreviations are used in this manuscript:

**ACC**	**Accelerations**
**AUC**	Area Under the Curve
**AU**	Arbitrary Units
**BMI**	Body Mass Index
**CV**	Cross-Validation
**DEC**	Decelerations
**DMI**	Decision-Making Index
**DT**	Decision Tree Classifier
**GB**	Gradient Boosting Classifier
**GNSS/GPS**	Global Navigation Satellite System/Global Positioning System
**HR**	Heart Rate
**HRmax/%HRmax**	Maximum Heart Rate/Percentage of Maximum Heart Rate
**HSR**	High-Speed Running Distance
**HID**	High-Intensity Distance
**KNN**	K-Nearest Neighbors Classifier
**MAE**	Mean Absolute Error
**MD**	Match Day
**MEI**	Motor Effectiveness Index
**ML**	Machine Learning
**MRS**	Maximum Running Speed
**OPI**	Overall Performance Indicator
**R^2^**	Coefficient of Determination
**RF**	Random Forest Classifier
**RMSE**	Root Mean Square Error
**ROC**	Receiver Operating Characteristic Curve
**RPE**	Rating of Perceived Exertion
**sRPE**	Session Rating of Perceived Exertion
**SD**	Standard Deviation
**SVM**	Support Vector Machine
**SSG**	Small-Sided Games
**TQR**	Total Quality Recovery
**U11/U13**	Under-11/Under-13 age categories
**Z1–Z5**	Heart rate intensity zones (Zones 1 to 5)
